# Highly efficient self-powered perovskite photodiode with an electron-blocking hole-transport NiO_x_ layer

**DOI:** 10.1038/s41598-020-80640-3

**Published:** 2021-01-08

**Authors:** Amir Muhammad Afzal, In-Gon Bae, Yushika Aggarwal, Jaewoo Park, Hye-Ryeon Jeong, Eun Ha Choi, Byoungchoo Park

**Affiliations:** Department of Electrical and Biological Physics, Kwangwoon University, Wolgye-Dong, Seoul, 01897 South Korea

**Keywords:** Solar energy and photovoltaic technology, Optical sensors, Imaging and sensing

## Abstract

Hybrid organic–inorganic perovskite materials provide noteworthy compact systems that could offer ground-breaking architectures for dynamic operations and advanced engineering in high-performance energy-harvesting optoelectronic devices. Here, we demonstrate a highly effective self-powered perovskite-based photodiode with an electron-blocking hole-transport layer (NiO_x_). A high value of responsivity (*R* = 360 mA W^−1^) with good detectivity (*D* = 2.1 × 10^11^ Jones) and external quantum efficiency (*EQE* = 76.5%) is achieved due to the excellent interface quality and suppression of the dark current at zero bias voltage owing to the NiO_x_ layer, providing outcomes one order of magnitude higher than values currently in the literature. Meanwhile, the value of *R* is progressively increased to 428 mA W^−1^ with *D* = 3.6 × 10^11^ Jones and *EQE* = 77% at a bias voltage of − 1.0 V. With a diode model, we also attained a high value of the built-in potential with the NiO_x_ layer, which is a direct signature of the improvement of the charge-selecting characteristics of the NiO_x_ layer. We also observed fast rise and decay times of approximately 0.9 and 1.8 ms, respectively, at zero bias voltage. Hence, these astonishing results based on the perovskite active layer together with the charge-selective NiO_x_ layer provide a platform on which to realise high-performance self-powered photodiode as well as energy-harvesting devices in the field of optoelectronics.

## Introduction

There is strong demand for high-performance photodetector devices owing to their favourable potential for both civilian and military use in light-detecting devices such as light-imaging sensors and applications such as night-time surveillance^1,2^. Specifically, light-harvesting materials and devices which convert photons to electricity have become the core components of several types of advanced optical technology^3^. For instance, silicon-based structures such as metal–oxide–semiconductors (MOS) are used in modern optoelectronic industries for the purposes of optical communications and are used in high sensitivity image processing cameras. However, serious breakdowns can occur in MOS-based photodetectors due to the high leakage current triggered by their thin oxide layers^4^.

Currently, instead of MOS, perovskite materials show numerous attractive characteristics, such as ambipolar transport, bandgap tuning, high external quantum efficiency (*EQE*), considerable optical absorption, and long charge carrier diffusion lengths, all of which form the main key parameters for optoelectronic devices, especially for photovoltaic (PV) cells and photodetectors^5,6^. Many complex perovskite composites can be perceived as striking substitutes to silicon-based photodetectors and imaging sensors. For example, smooth, highly crystallised, and pinhole-free Sn- and Pb-rich binary perovskite composite films can be critical material systems in the effort to realise efficient photodetectors^7,8^. Such perovskite-based self-powered photodetectors show an extraordinary responsivity (*R*) value of 0.2 A W^−1^ at 940 nm and a fast response time of 2.27 μs, exceeding those of most silicon-based imaging sensors. Specifically, perovskite composites of CH_3_NH_3_PbX_3_ (X = Br, Cl, and I) have also attracted much attention in relation to optoelectronic technology given their potential for enhanced performance, with one example being light-harvesting devices^9,10^. Thus, Hu et al. fabricated a lateral type of photodiode based on perovskite (CH_3_NH_3_PbI_3_) and measured the responsivity *R* and *EQE* at a high bias voltage (*V*_bias_ = 2 V)^11^. Further, Chen et al. demonstrated perovskite photodiode which utilise graphene as an electrode and found relatively small values of *R* (22 mA W^−1^) and detectivity (*D*) $\left( {3.55 \times 10^{9} \,{\text{Jones}}} \right)$^12^. Zhou et al. established a perovskite photodiode with *R* values of approximately 198 mA W^−1^^13^. However, the prevailed fabrication methods and strategies reported thus far will inevitably lead to grain boundary issues and large variations in the morphologies of the resultant perovskite active layers in the devices. Further, the relatively small values of the responsivity *R* and detectivity *D* of perovskite photodiodes and their comparatively large dark current densities are the major hurdles to overcome to realise high-performance devices. Moreover, suitable materials capable of forming a good interface with the perovskite active layer remain elusive in the field of perovskite-based photodiode during the effort to achieve high *R* and *D* values as well as fast response times. Thus, the device performance capabilities of recent perovskite-based photodiode remain insufficient.

Meanwhile, researchers remain motivated to realise an innovative photodiode exhibiting astonishing performance and a high *R* value using other functional layers as hole transport layers (HTLs) and electron transport layers (ETLs) together with hybrid heterostructures exhibiting tailored, novel, and improved characteristics^14–16^. In order to achieve excellent device performance with regard to photoconductivity, the proper selection of the HTLs is crucial, not only to decrease the dark current density but also to enhance and promote perfect light absorption of the perovskite active layer in the wide visible region^17–19^. Metal-oxide (MO) or ternary MO nanoparticles (NPs) synthesised by a solution process are favourable for unlocking their potential in solar cells as HTLs given their low cost, good stability, and promising optical characteristics. However, exotic organic ligands adopted for the purpose of ensuring a small size and a nano-dispersion are associated with poor conductivity, which thus impedes their use in electrical applications^20^. Well-dispersed NiCo_2_O_4_ ternary MO NPs synthesised by a unique method without exotic ligands have been used successfully as a HTL in PVSCs. The pinhole-free films of NiCo_2_O_4_ NPs facilitated the formation of large grains of perovskite films. As a result, the power conversion efficiency (PCE) was enhanced to 18%, with promising stability^21^. Other ternary MO NPs of In-doped CuCrO_2_ have also been proposed as an efficient HTL material system. Interestingly, the PCE of the PVSCs with the In:CuCrO_2_ HTL was increased to 20.5% with good repeatability and photostability^22^. Thus, new approaches are mandatory for the realisation of excellent perovskite photodiode using heterostructures of organic and inorganic materials combined with perovskite materials.

Recently, among inorganic semiconductor thin films, nickel oxide (NiO_x_) has been considered as a crucial building block for modern optoelectronic devices owing to its versatile physical and chemical features along with its appropriate band structure. Specifically, NiO_x_ has been used in organic and perovskite PV cells as a HTL owing to its efficient chemical and thermal stability, outstanding hole-transport characteristics, large band gap (> ~ 3.7 eV), the ability to control the valence-band energy level effectively, and its deep valence band position (> 5.2 eV)^23–25^. Chen et al. used a NiO_x_ HTL synthesised by a sputtering method at a low temperature to devise inverted-type perovskite PV cells, achieving a PCE of 11.6%^26^. Zhu et al. introduced a solution-processed NiO_x_:PbI_2_ nanocomposite structure which operated at room-temperature to assist with compact and crystalline MAPbI_3_ film growth, which is critical during the fabrication of effectual photodetectors. This nanocomposite served as an electron-blocking, hole-extracting, and passivation layer and ultimately suppressed the dark current, resulting in improved photo characteristics, such as better detectivity and faster response times^27^. More recently, a thin interlayer of NiO_x_, synthesised by a comparatively simple solution-processed method demonstrated tunable work functions (5.0 – 5.6 eV) and efficient hole conductivity outcomes^28,29^. A thin layer of NiO_x_ as a HTL in perovskite PV cells was also shown to hinder the penetration of water and oxygen into a device effectively^30^. Zin et al. proposed a simple method by which to synthesise a NiO_*x*_ layer and used it as a HTL in inverted perovskite PV cells. Their device showed greatly improved values of PCE (~ 20.2%)^24,31^. Particularly, a thin NiO_*x*_ film showed enhanced charge carrier density and conductivity through improvements of the interfacial charge extraction, thus reducing the interface trap density and providing better energy level alignment with the perovskite layer in perovskite solar cells^32^.

Inspired by these remarkable characteristics of NiO_x_ layers, a heterostructure approach which involves the assembly of a halide perovskite with the inorganic semiconducting material of NiO_x_ may represent an alternative means by which to design photodiode that are anticipated to have enhanced photosensitivity. In this work, a high-performance and unique perovskite photodiode with a HTL of NiO_x_ exhibiting a high responsivity value is reported. We designed a self-powered hybrid perovskite-based photodiode structure that suppresses the dark leakage current and enhances the performance of the perovskite photodiode. The effects of the HTL of NiO_x_ in the perovskite photodiode on the device performance and limitation factors are investigated by evaluating the optoelectronic characteristics of the photodiode. We measured the optoelectronic characteristics of the photodiode device under different power levels and wavelengths of incident laser light to obtain the *R*, *D*, and *EQE* values at a given bias voltage. Further, we took temporal photoresponse measurements to determine the response time (rise and decay time) characteristics. We also verified the *EQE* values through incident-photon-to-electron conversion efficiency (IPCE) measurements and confirmed the degree of self-consistency in the results. Moreover, we measured the characteristics of reference devices with poly (3, 4-ethylene dioxythiophene) polystyrene sulfonate (PEDOT:PSS) as a HTL in order to compare and support our results, showing that NiO_x_ as a HTL exhibits much improved photodiode performance as compared to the conventional HTL of PEDOT:PSS.

## Results

**Figure 1:**
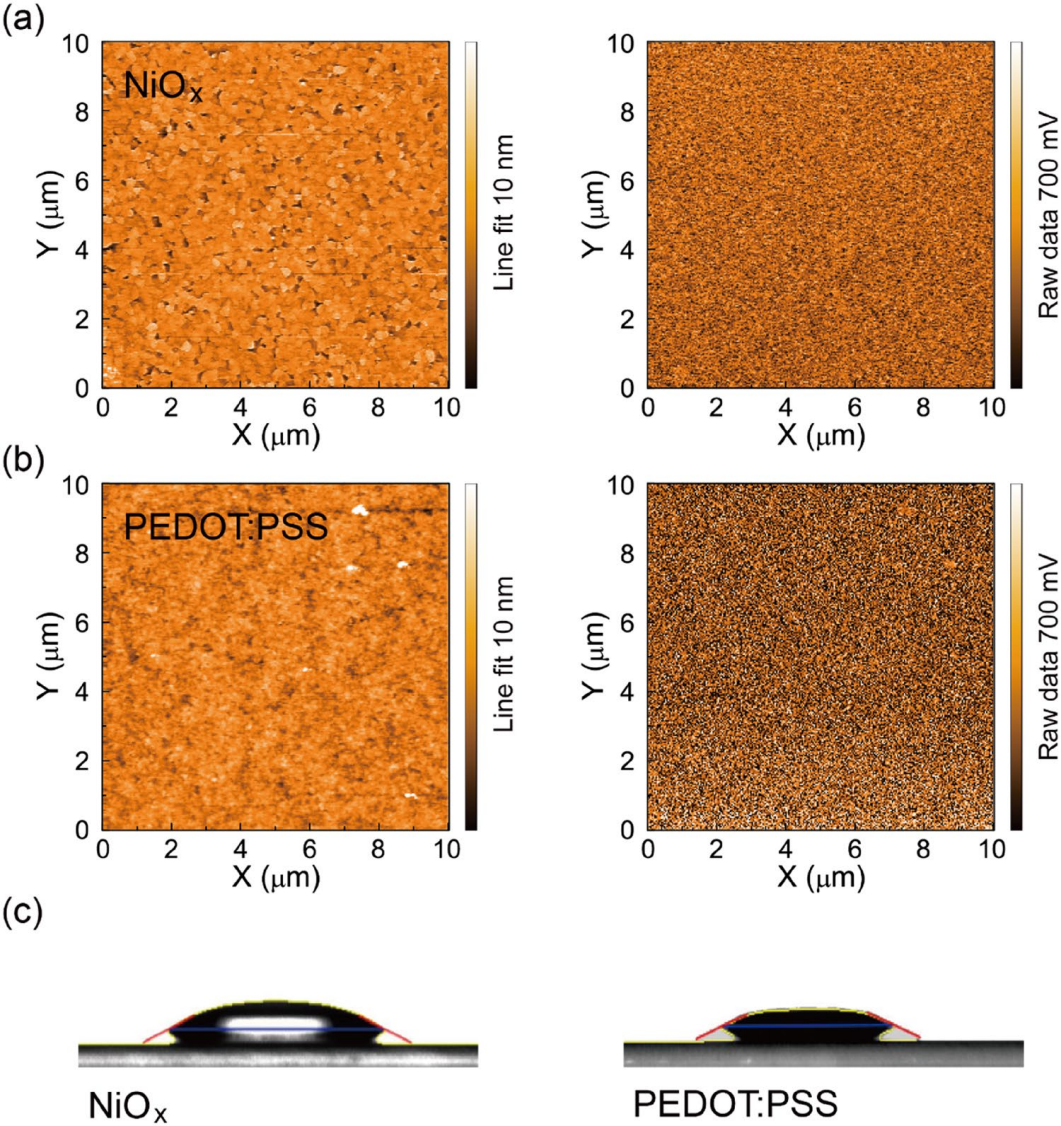
AFM (left) and KPFM (right) images of a sample NiO_x_ layer (**a**) and a reference PEDOT:PSS layer (**b**), and (**c**) water contact angles of the NiO_x_ (left) and PEDOT:PSS (right) layers.

Initially, in order to understand the surface properties of the fabricated sample NiO_x_ and reference PEDOT:PSS layers, atomic force microscopy (AFM), Kelvin probe force microscopy (KPFM), and contact angle measurements were utilised to characterise the layers, especially with regard to their surface roughness and surface potential levels. Figure 1a,b correspondingly show the observed results of the AFM topologies and KPFM surface potential maps of the NiO_x_ and PEDOT:PSS layers coated onto indium tin oxide (ITO) substrates. The nearly identical roughness values of the NiO_x_ and PEDOT:PSS layers were approximately 0.73 nm. Thus, the fabricated NiO_x_ layer on the ITO substrate is quite flat and uniform. From the obtained KPFM surface potential maps, we also determined the Fermi levels of the NiO_x_ layer^33^, finding a value of approximately 5.1 eV, slightly higher than that (5.0 eV) of the PEDOT:PSS layer.

We also measured the water contact angles of the NiO_x_ layer and compared these outcomes with those of the reference PEDOT:PSS layer, as shown in Fig. 1c. The observed water contact angles were 16.1° ± 2.8° for the NiO_x_ layer and 13.4° ± 1.4° for the PEDOT:PSS layer, indicating that the NiO_x_ surface is more hydrophobic than the PEDOT:PSS layer. Thus, the relatively non-wetting NiO_x_ surface may improve the film formation of the active perovskite layer and facilitate uniform and continuous perovskite film morphologies with large crystalline domains^34,35^.

**Figure 2:**
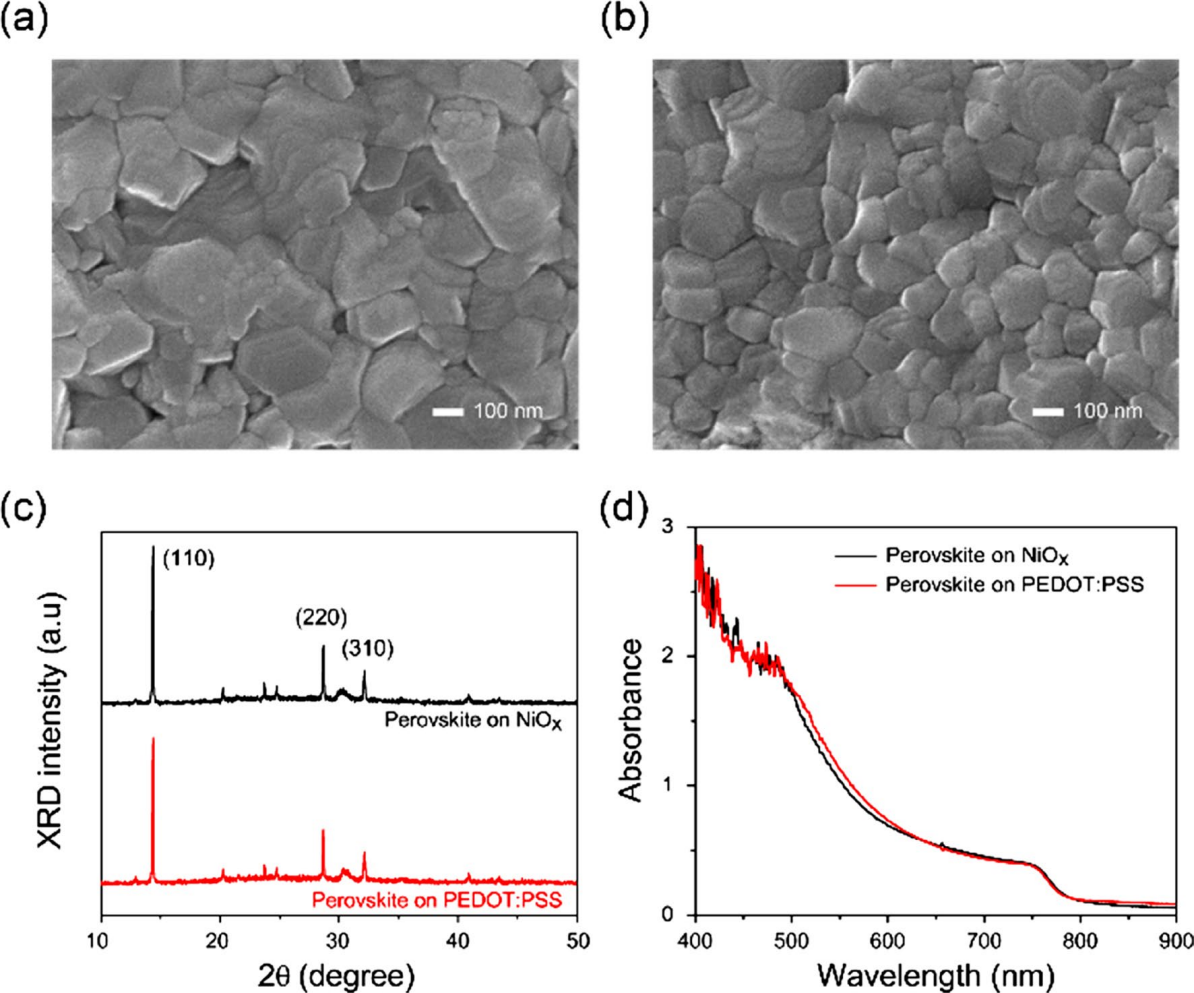
(**a**) SEM image of a perovskite film on the NiO_x_ layer, (**b**) SEM image of a perovskite film on the PEDOT:PSS layer, (**c**) XRD spectra of the perovskite films on the NiO_x_ and PEDOT:PSS HTLs, and (**d**) optical absorbance spectra of the perovskite films on the NiO_x_ and PEDOT:PSS HTLs.

Next, we examined the effects of the NiO_x_ and PEDOT:PSS layers on the formation of a light harvester, in this case a perovskite layer (CH_3_NH_3_PbI_3_, MAPbI_3_). Here, the perovskite layers were prepared using a toluene-assisted rapid-crystalline technique^36^, to attain a uniform, continuous, flat, and full-coverage film, as mentioned in the “Experimental section”. The thickness of the perovskite layer was fixed at 250 nm in all layers and devices in this study, as the device performance of a perovskite photodiode can be influenced by the perovskite film thickness^37^. To compare the film quality levels of the fabricated perovskite layers on the NiO_x_ and PEDOT:PSS underlying layers as HTLs, the surface morphologies of the perovskite films on the NiO_x_ and PEDOT:PSS HTLs were characterised by scanning electron microscopy (SEM). Figure 2a,b show surface SEM images of the CH_3_NH_3_PbI_3_ perovskite layers on the NiO_x_ and PEDOT:PSS HTLs, respectively. Notably, the grain size $\left( { \approx 254\,{\text{nm}}} \right)$ of the perovskite film on the NiO_x_ HTL greatly exceeds that $\left( { \approx 166\,{\text{nm}}} \right)$ on the PEDOT:PSS HTL. This result verifies that the relatively non-wetting NiO_x_ surface suppresses perovskite nucleation sites, which facilitates the growth of larger grains^38^. The large grain size of the perovskite film can reduce the recombination rate of the charge carriers due to the decreased charge trap density^34^.

As depicted in Fig. 2c, the perovskite films on the NiO_x_ and PEDOT:PSS HTLs were also characterised by X-ray diffractometry (XRD). Robust and major Bragg peaks were observed at 14.3°, 28.6°, and 32.1°, corresponding to the (110), (220), and (310) planes of the MAPbI_3_ perovskite film given its orthorhombic crystal structure^34^. On the NiO_x_ HTL, we observed a slight improvement in the XRD peak intensity levels of the perovskite film as compared to those on the PEDOT:PSS HTL, also implying the larger grains with high crystallinity of the perovskite film. Note that there was no significant change in the XRD peak ratios, implying that the different HTLs did not cause any substantial change in the crystal orientations of the perovskite films. Figure 2d shows the optical absorption spectra of the perovskite films on the NiO_x_ and PEDOT:PSS HTLs. We observed that the absorption spectra of the perovskite layers in both cases on the HTLs were nearly identical to each other^34^. It was also noted that the optical transmittance levels of the NiO_x_ layer are comparable but slightly lower in the visible range compared to those of the PEDOT:PSS layer (Fig. S1).

**Figure 3:**
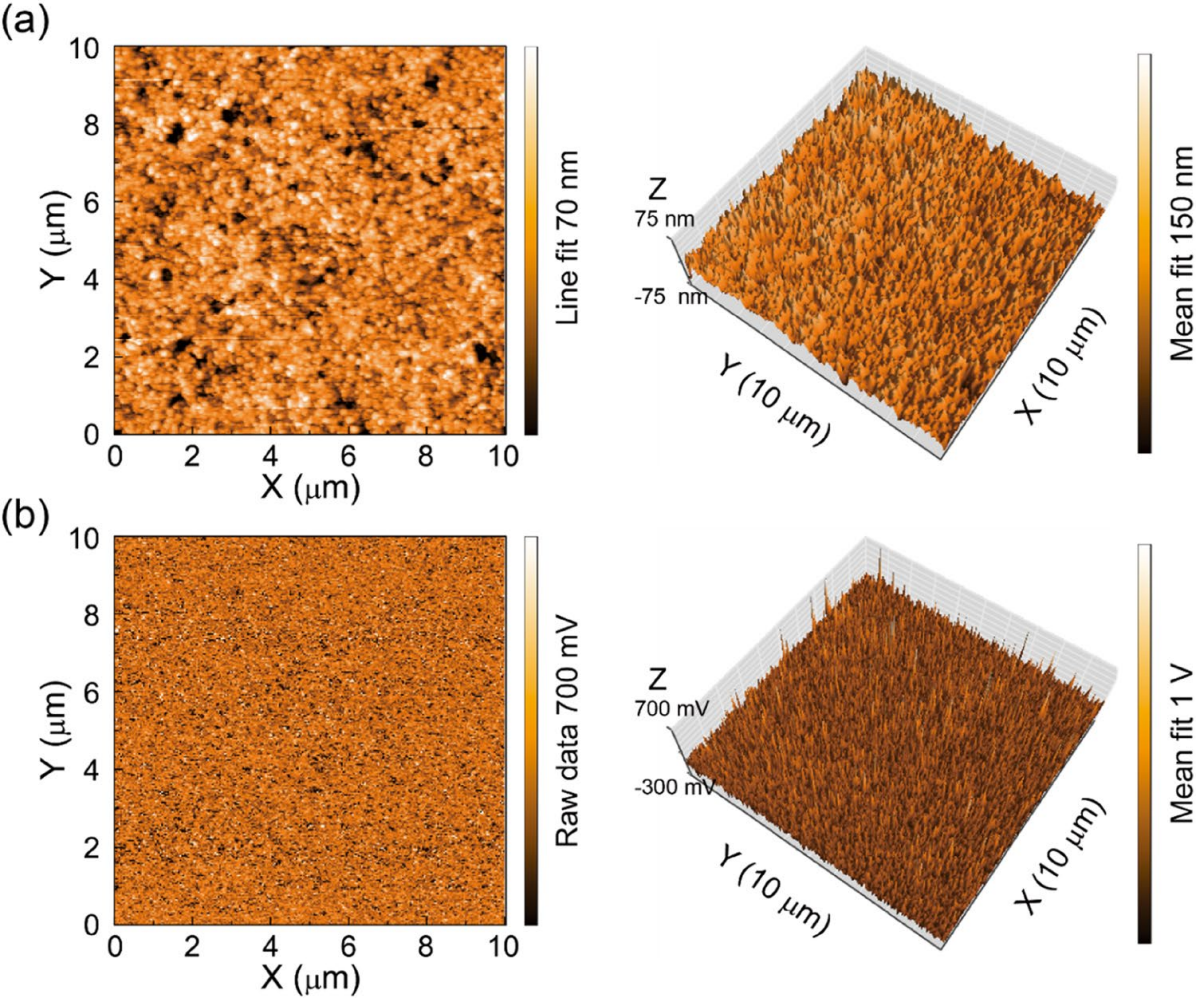
(**a**) AFM image of the perovskite film on the NiO_x_ HTL (left) with a three-dimensional plot (right) of the perovskite film, and (**b**) KPFM image of the perovskite film on the NiO_x_ HTL (left) with a three-dimensional plot (right).

Next, in order to examine the effects of the NiO_x_ and PEDOT:PSS HTLs on the surface roughness and energy level of the perovskite layer, AFM and KPFM were used to characterise the perovskite layers on the NiO_x_ and PEDOT:PSS HTLs. Figures 3a and b show the results of observations of the AFM topologies and the KPFM surface potential maps of the perovskite film on the NiO_x_ HTL, respectively. For comparison, AFM images and KPFM surface potential maps of the perovskite film on the PEDOT:PSS HTL are shown in Fig. S2. The roughness values of the perovskite films on the NiO_x_ and PEDOT:PSS HTLs were approximately 8.5 and 9.7 nm, respectively. These results indicate that the active MAPbI_3_ perovskite layer formed on the NiO_x_ HTL is more continuous, flat, and uniform than that on the PEDOT:PSS HTL. We also determined the Fermi levels of the perovskite layers on the HTLs using the obtained surface potentials^33^, finding that they were approximately 5.1 eV. It is noted that the Fermi level of the NiO_x_ HTL is closer to that of the perovskite film as compared to that of the PEDOT:PSS HTL. Thus, we considered that the interface quality and affinity between the perovskite layer and the NiO_x_ HTL are better than those between the perovskite layer and the PEDOT:PSS HTL due to the good crystallinity with large grains, low surface roughness, and a small difference in the Fermi level of the perovskite layer on the NiO_x_ layer, all of which may promote the performance of perovskite photodiodes^39,40^.

**Figure 4:**
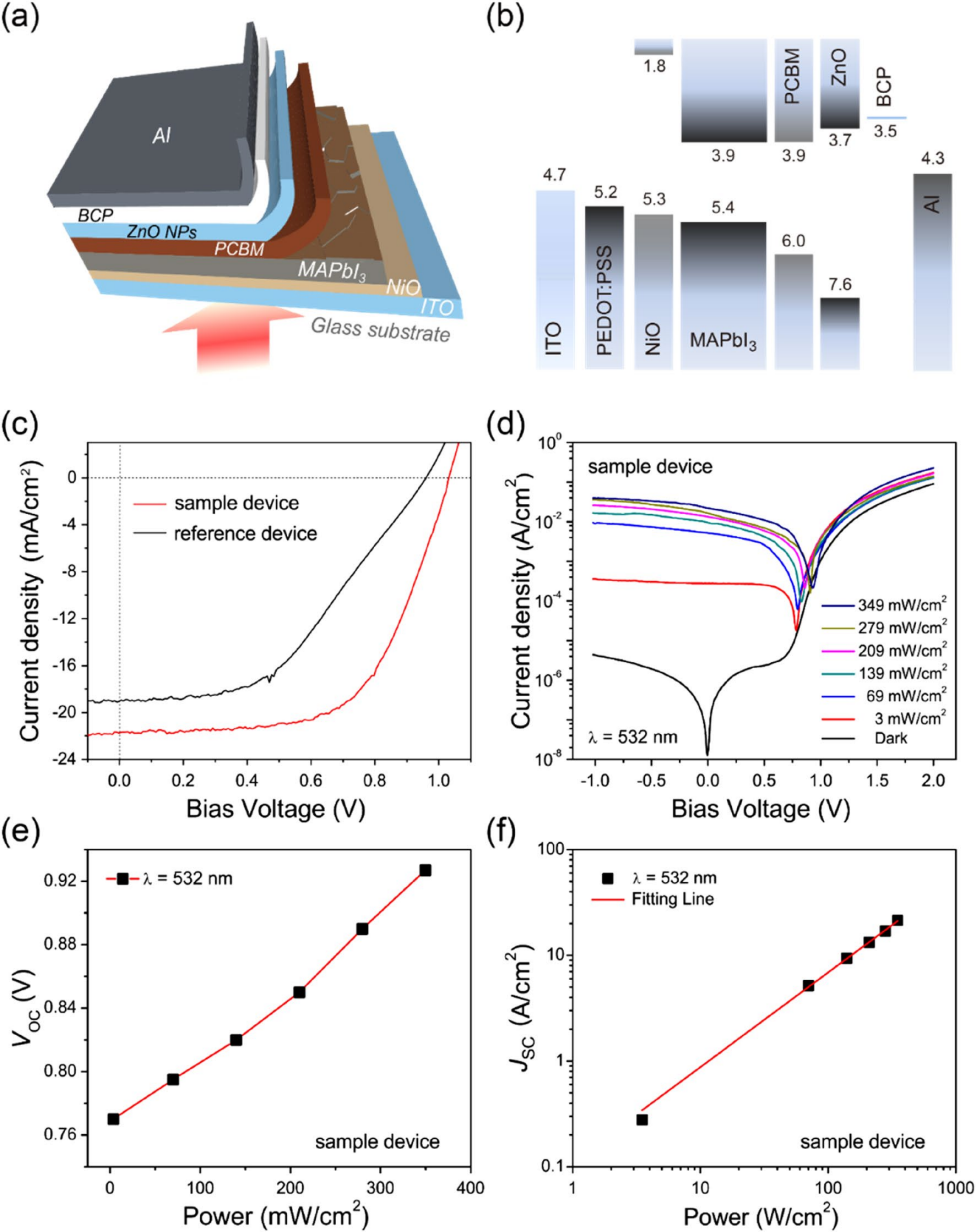
(**a**) Schematic diagram of a self-powered perovskite photodiode with NiO_x_ as an electron-blocking and HTL, (**b**) energy band diagram of the self-powered perovskite photodiode, (**c**) photovoltaic measurements of the perovskite photodiodes under an AM1.5G illumination source, (**d**) photocurrent density of the perovskite photodiode with the NiO_x_ HTL as a function of the bias voltage for several different intensity levels of incident light with a wavelength of 532 nm, (**e**) open-circuit voltage (*V*_oc_) of the photodiode with the NiO_x_ HTL as a function of the input power of the incident laser light, and (**f**) short-circuit current density (*J*_sc_) of the photodiode with the NiO_x_ HTL as a function of the input power of incident light (532 nm) at zero bias voltage.

Subsequently, we investigated the device performance of a self-powered perovskite photodiode. A schematic illustration of the self-powered perovskite photodiode is presented in Fig. 4a. In the structure, ITO is used as the anode material, NiO_x_ serves as the HTL, the perovskite layer (CH_3_NH_3_PbI_3_) functions as the active layer, phenyl-C61-butyric acid methyl ester (PCBM_60_) and ZnO NPs form the ETLs, bathocuproine (BCP) is used as a buffer layer for a reduction of the nonradiative recombination of excitons, and Al is utilised as the cathode (for details, see the “Experimental section”).

Figure 4b shows the energy band diagram of a photodiode with NiO_x_ or PEDOT:PSS as the HTL. As shown in the fig., it is clear that the NiO_x_ HTL has a higher valence band maximum (VBM) value (5.3 eV) compared to that (5.2 eV) of the PEDOT:PSS HTL^41,42^. Thus, the NiO_x_ HTL provides a more favourable alignment of the energy level with respect to the VBM of the perovskite active layer. The high value of the VBM of the NiO_x_ HTL would decrease the energy barrier, which may in turn increase the build-in potential (*V*_bi_) and facilitate improved hole transportation, consequently leading to a larger value of the open-circuit voltage (*V*_oc_)^24,34,41^. In order to estimate the PV characteristics of the fabricated self-powered perovskite photodiodes, the photocurrent density–voltage (*J*–*V*) characteristics of the photodiodes were measured under the AM1.5G illumination source. Figure 4c shows the observed *J*–*V* characteristics of the sample perovskite photodiode with the NiO_x_ HTL (hereafter, the sample device) and of the reference photodiode with the PEDOT:PSS HTL (hereafter, the reference device). The estimated PCE value from the *J*–*V* curve was 13% in the sample device with an open-circuit voltage *V*_OC_ of 1.03 V, a short-circuit current density (*J*_SC_) of 21 mA cm^−2^, and a fill factor (*FF*) of 61%; the PCE in this case is much higher than the PCE value of 8% in the reference device with *V*_oc_ = 0.95 V, *J*_sc_ = 19 mA cm^−2^, and *FF* = 45%. These PV results are consistent with previous results for perovskite PV cells^26,43,44^. Thus, it is obvious that the perovskite photodiodes based on the NiO_x_ HTL exhibit improved PV performance. Note that the increased *V*_oc_ value of the sample device is mainly due to the high VBM of the NiO_x_ HTL, which implies an increase in the built-in potential^29^. We also took measurements via forward and backward scans of the photodiode devices studied here (Fig. S3) to augment our discussion of the hysteresis in the *J*–*V* scans of the perovskite devices^45–47^. We calculated the hysteresis index (HI) using the relationship of ${\text{HI}} = \frac{{J_{RS} \left( {0.8V_{oc} } \right) - J_{FS} (0.8V_{oc} ))}}{{J_{RS} \left( {0.8V_{0c} } \right)}}$, where *J*_RS_(0.8*V*_OC_) and *J*_FS_(0.8*V*_OC_) represent the photocurrent densities at a bias voltage of 0.8 *V*_OC_ for the reverse and forward scans, respectively^47^. The estimated values of HI for the photodiodes with the NiO_x_ and PEDOT:PSS HTLs are 0.06 and 0.60, respectively, which indicates that the sample photodiode with the NiO_x_ HTL shows negligible hysteresis. Thus, the much smaller value of HI for the photodiode with the NiO_x_ HTL than for that with the PEDOT:PSS HTL is mainly owing to the high crystallinity of the perovskite layer and rapid charge extraction by the NiO_x_ HTL^45–47^.

At this point, we focus our attention on the photodiode performance of the sample device fabricated with NiO_x_. Figure 4d shows the current density as a function of the bias voltage (*J*–*V*) at different input power levels (0 – 349 mW cm^−2^) of incident laser light having a wavelength of $\lambda = 532 {\text{nm}}$. We swept the voltage from − 1.0 to + 2.0 V and measured the current density *J*. Both the forward and reverse current density levels increase gradually as the input power of the incident light increases.

Meanwhile, asymmetric electrodes (ITO and Al) of the fabricated perovskite photodiodes generate the built-in potential *V*_bi_ that drives the operation of the photodiodes, even under zero external bias voltage, *i.e.*, a self-powered condition. Figure 4e,f show the variations in *V*_oc_ and the short-circuit current density *J*_sc_, as a function of the input power of the incident laser light (532 nm), respectively, for the sample device with NiO_x_. As shown in the figures, *V*_oc_ and *J*_sc_ increase gradually to 0.92 V and 23 mA cm^−2^, respectively, as the input power of the incident light increases from 0 to 349 mW cm^−2^. Here, $J_{sc}$ is increased considerably, likely due to the large number of photo-excited charge carriers separated under the high built-in potential *V*_bi_. The *V*_oc_ and *J*_sc_ values for the reference device with PEDOT:PSS are also correspondingly shown in Figs. S4 and S5. As compared in these figures, higher values of *V*_oc_ and *J*_sc_ for the sample device were clearly observed. This result stems from the better electron-blocking and hole-transporting capabilities of the NiO_x_ HTL. It is also noted that *J*_sc_ reveals linear dependence on the input power of the incident light (Figs. 4f and S8). This dependence of *J*_sc_ can be analysed using the following relationship^48^,

1 $$J_{sc} = cP^{\theta } ,$$

where *c*, *P*, and $\theta$ are a proportional constant, the input power intensity of the incident laser light, and the power-law index, respectively. With best-fit parameters, the obtained value of $\theta$ for the sample device with NiO_x_ is 0.89, which is close to 1.0 for an ideal photodiode with a low trap state junction and which is noticeably higher than that ($\theta$ = 0.69) for the reference device with PEDOT:PSS (Fig. S5). Such a relatively large $\theta$ value for the sample device indicates that a small number of trap states exist in the perovskite layer on the NiO_x_ HTL, which is valuable for the realisation of high photo-sensing ability through the efficient collection of a large number of photo-excited charge carriers.

**Figure 5:**
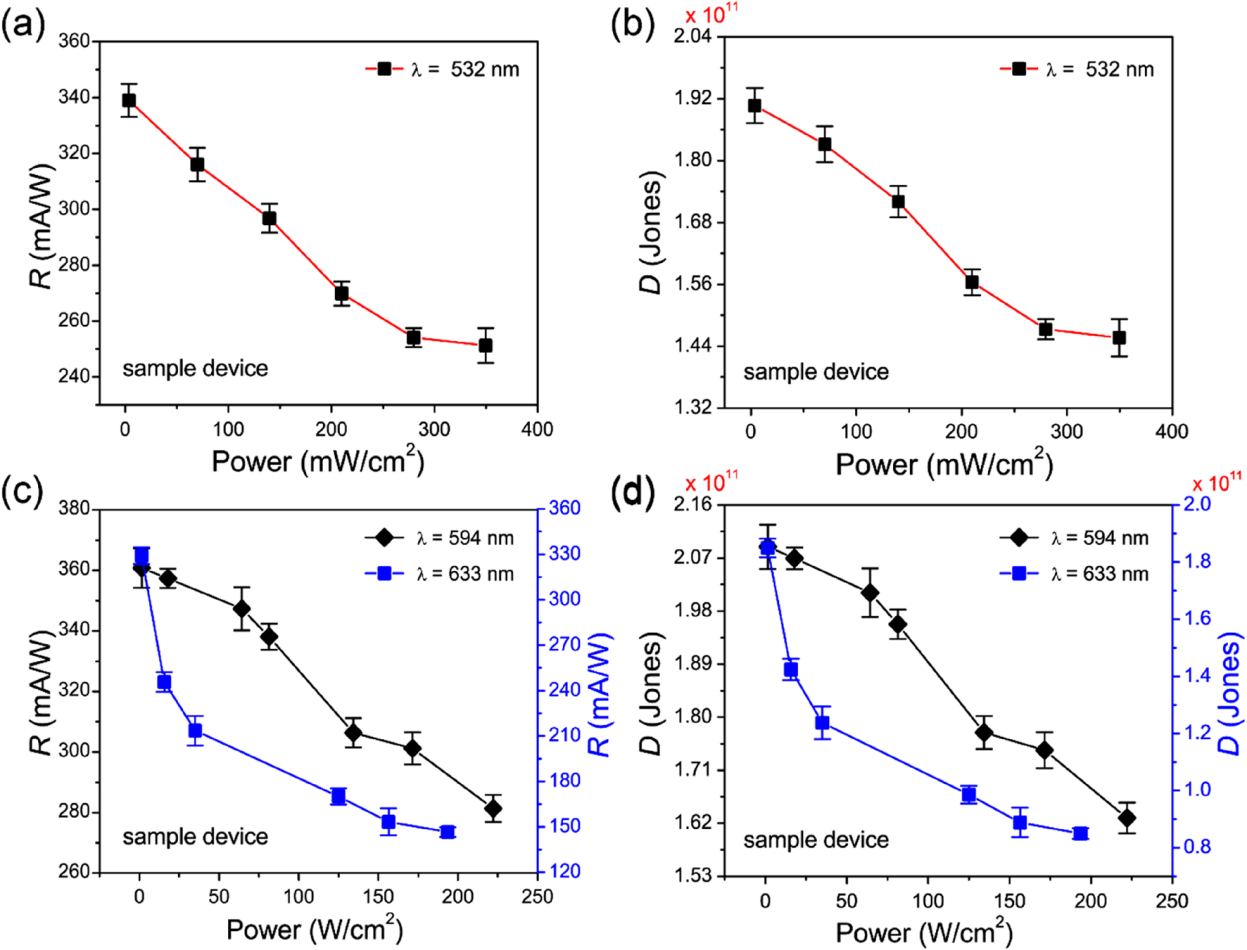
Responsivity *R* (**a**) and detectivity *D,* (**b**) of the perovskite photodiode with the NiO_x_ HTL as a function of the input power of incident light (532 nm) at zero bias voltage, and comparison of the responsivity *R,* (**c**) and detectivity *D,* (**d**) of the photodiode as a function of the input power of incident light with different wavelengths (594 and 633 nm) at zero bias voltage.

Next, to evaluate *R* and *D* of the perovskite photodiode at zero bias voltage (*V*_bias_ = 0 V, self-powered condition), we used the following relationship^49^,

2 $$R = J_{PH} /PA,$$

where $J_{PH}$ is the net photocurrent density $\left( {J_{PH} = J_{light} - J_{dark} } \right)$ and *A* denotes the illuminating junction area of the photodiode for the incident laser light. Figure 5a shows the *R* value, estimated from the current density data (shown in Fig. 4d), as a function of the input power of incident laser light (532 nm) for the sample device. The highest estimated *R* value of the sample device is 340 mA W^−1^ at zero bias voltage (self-powered), which is much higher as compared to previously reported values, mainly due to the efficient suppression of the dark leakage current. Moreover, the value of *R* increases to 440 mA W^−1^ when the bias voltage *V*_bias_ = − 1.0 V. We also estimated the detectivity *D* value of the sample device using the following relationship^50^,

3 $$D = \frac{R}{{\sqrt {2qJ_{dark} } }},$$

where $q$ denotes the charge of the electron. Figure 5b shows the *D* values obtained for several input power levels of incident laser light (532 nm). The highest estimated *D* value of the sample device with NiO_x_ is approximately $1.9 \times 10^{11}$ Jones, which is also much higher than earlier values reported in the literature.

Additionally, we measured the *R* and *D* values of the sample device with NiO_x_ with incident laser light at wavelengths of 594 and 633 nm to quantify the wavelength selectivity of the photodiode, as shown in Fig. 5c,d, respectively. In this case, we also obtained high corresponding *R* values of 360 and 330 mA W^−1^ with improved *D* values of $2.08 \times 10^{11}$ and $1.8 \times 10^{11}$ Jones under incident laser light illumination with wavelengths of 594 and 633 nm, respectively, at zero bias voltage. These results clearly demonstrate the highly efficient self-powered operation of the perovskite photodiode studied here.

**Figure 6:**
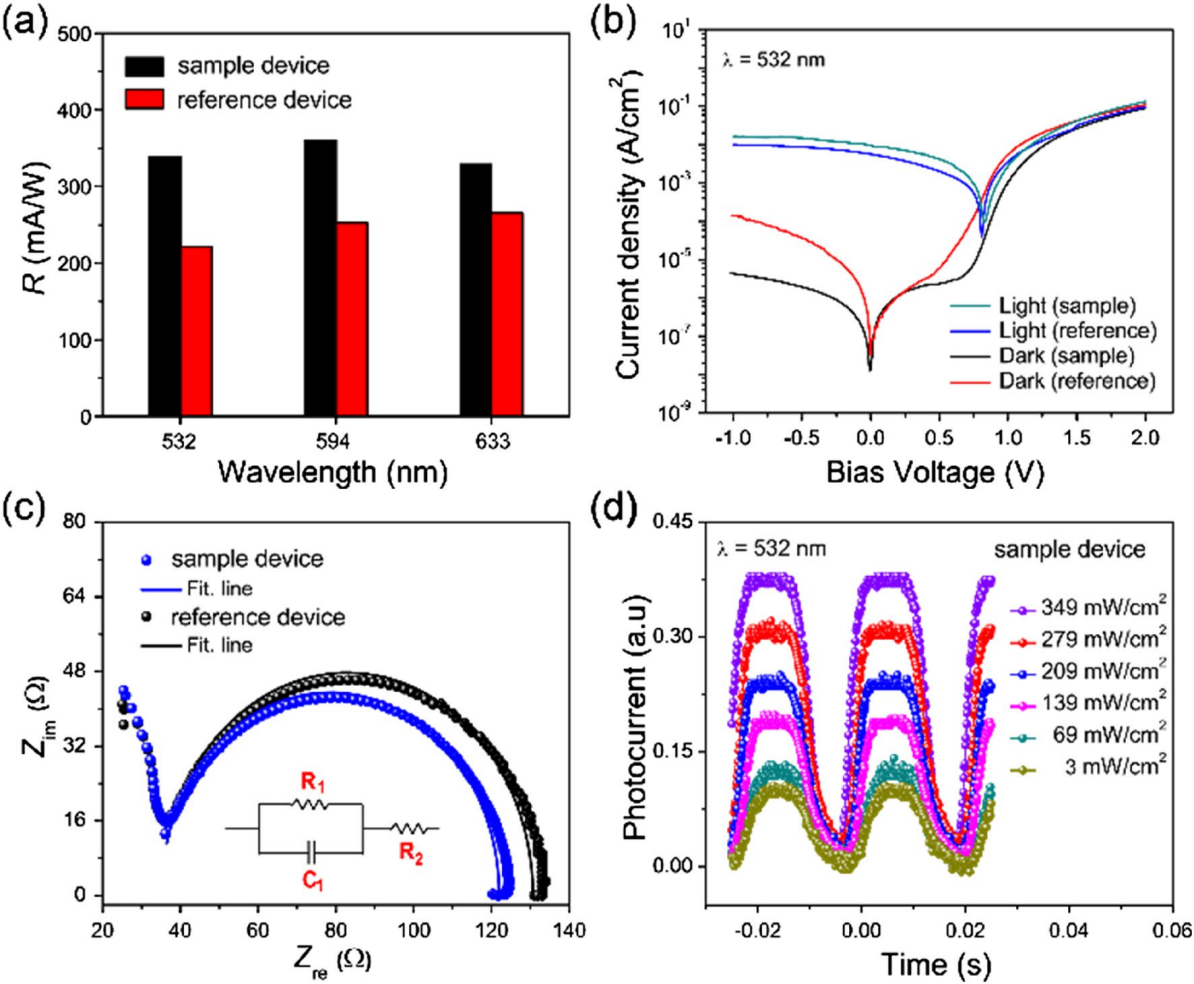
(**a**) Comparison of *R* values for the perovskite photodiodes with NiO_x_ and PEDOT:PSS as HTLs, (**b**) dark- and photo-current densities of the photodiodes with NiO_x_ and PEDOT:PSS as HTLs under illumination of incident light (532 nm), (**c**) impedance spectra (IS) of the photodiodes with the NiO_x_ or PEDOT:PSS HTLs at zero bias voltage, and (**d**) temporal photoresponses of the photodiode with the NiO_x_ HTL for several input power levels of incident light (532 nm) at zero bias voltage when turning the incident light on and off.

Figure 6a shows comparisons of the *R* values obtained from the sample and reference devices in three incident light with different wavelengths. Clear differences were noted in *R* values, and the *R* value is enhanced from 240 to 360 mA W^−1^ at 594 nm when NiO_x_ is introduced to replace the conventional PEDOT:PSS as the HTL. This outcome provides clear evidence that the sample device based on the NiO_x_ HTL exhibits superior responsivity *R* relative to the reference device based on the conventional PEDOT:PSS HTL. We also measured the stability of the photodiodes studied here with the NiO_x_ and PEDOT:PSS HTLs (Fig. S6). As shown in the figure, the device with NiO_x_ is much more stable as compared to that with PEDOT:PSS^25,51^. Thus, it is clear that our self-powered perovskite-based photodiode with NiO_x_ is promising for highly sensitive photodiodes requiring low energy consumption levels.

## Discussion

In order to understand the cause of such high responsivity of the sample device with NiO_x_, the dark and photo *J*–*V* characteristics were compared and analysed, as shown in Fig. 6b. In this figure, we also compare the *J–V* curves of the reference device with PEDOT:PSS. In a dark condition, we observed clear diode behaviour with a high rectification ratio of $2.0 \times 10^{4}$ for the sample device. This rectification ratio is nearly 27 times higher than that ($7.5 \times 10^{2}$) for the reference device. Moreover, the sample device with NiO_x_ clearly shows relatively low leakage current compared to the reference device with PEDOT:PSS. The dark current density is suppressed in the sample device to $7.84 \times 10^{ - 6}$ mA cm^−2^, a much smaller value as compared to that ($2.84 \times 10^{ - 5} \,{\text{mA}}\,{\text{cm}}^{ - 2}$) of the reference device at zero bias voltage. This reduction in the dark current density confirms the low leakage current in the perovskite photodiode with NiO_x_^27^. Thus, it is clear that the perovskite photodiode based on the NiO_x_ HTL exhibits much better diode characteristics than the reference device with the conventional PEDOT:PSS HTL. Further, the sample device shows higher photocurrent density levels under incident laser light in both the forward and reverse regions as compared to the results of the reference device. The photocurrent density of the sample device increases to 7.5 mA cm^−2^ under incident laser light (532 nm) with an input power of 349 mW cm^−2^ at zero bias voltage. Therefore, we considered that the relatively low leakage current in the dark and the increased photocurrent outcomes of the sample device with NiO_x_ may promote the improved device performance through the realisation of a leakage-free photodiode.

To clarify the remarkable effect of the NiO_x_ HTL on the high responsivity of the perovskite photodiode, the dark current flows of the devices (Fig. 6b) are analysed using the following relationship for the Shockley diode in a single-junction device^52^,

4 $$J = J_{0} \left[ {\exp \left( {\frac{qV}{{nKT}}} \right) - 1} \right],$$

where $J_{0}$, *n*, *K*, and *T* represent the saturation current density, ideality factor, Boltzmann’s constant, and temperature, respectively. For an ideal diode (p–n junction), the ideality factor *n* is expected to be equal to 1.0 without charge carrier trapping^53^. Thus, the ideality factor *n* can be a key parameter to estimate the suppression of the recombination rate in perovskite photodiodes. From the analyses of the dark currents, the estimated values of *n*s are 1.1 and 1.7 for the sample and reference devices, respectively. The smaller value of *n* for the sample device as compared to that for the reference device stems from the reduced number of monomolecular recombinations^53^. To verify this reduction of recombinations, we also estimated the recombination resistance $R_{rec}$ for the charge carriers using the following relationship^54^,

5 $$R_{rec} = \frac{{V_{e} }}{{J_{sc} }}\left( {e^{{q\left( {\frac{{V_{oc} - V_{e} - B}}{nKT}} \right)}} } \right) ,$$

where $V_{e}$, and $R_{s}$ represent the effective voltage, the minimum voltage used to extract *n* from the *J*–*V* curves, and the series resistance, respectively. Based on the above relationship, we obtained a higher value of $R_{rec} \left( { \approx 105 \Omega } \right)$ for the sample device with NiO_x_ as compared to that $\left( { \approx 79 {\Omega }} \right)$ for the reference device with PEDOT:PSS. Thus, the higher $R_{rec}$ for the sample device verifies higher resistivity to carrier recombinations in the photodiode with the NiO_x_ HTL^54,55^.

To clarify and examine the defect states and trap recombination at the HTL/perovskite interface, the space-charge limited current model is employed. The trap-state density (*n*_trap_) can be calculated using the following relationship^56^,

6 $$n_{trap} = \frac{{2\varepsilon \varepsilon_{o} V_{TFL} }}{{eL^{2} }},$$

where ε, ε_0_, *V*_TFL_, *e*, and *L* correspondingly represent the relative dielectric constant of perovskite, the dielectric constant of a vacuum, the trap-filling limited voltage, the elementary charge, and the thickness of the perovskite film. The value of *V*_TFL_ can be derived from the *J–V* characteristics of reference and sample photodiode devices in a dark condition (Fig. S7). The *V*_TFL_ values of the devices with the NiO_x_ and PEDOT:PSS HTLs are 0.42 and 0.74 V, respectively. Thus, the estimated *n*_trap_ values of the devices are 1.83 × 10^15^ cm^−3^ for the reference device with the PEDOT:PSS HTL and 1.12 × 10^15^ cm^−3^ for the sample device with the NiO_x_ HTL, clearly demonstrating fewer trap recombinations at the NiO_x_/perovskite interface^20,57^.

Next, the reduction of charge recombination of the sample device was analysed further on the basis of the open-circuit voltage *V*_oc_ because all of the photo-excited charge carriers will recombine within the device at the end at the open-circuit condition^36,58^. By linear least squares fitting of the *V*_oc_ data (Fig. 4e), slopes of $1.2 K_{B} T/q$ and $1.4 K_{B} T/q$ were attained for the sample and reference devices, respectively. In principle, when the slope is equal to $1.0{ }K_{B} T/q$, the device operates without trapping charge carriers or is governed by bimolecular recombinations, and the active layer is considered as having recombinations free of electrons and holes; when the slope exceeds $1.0{ }K_{B} T/q$, monomolecular Shockley–Read–Hall (SRH) recombinations are involved^53^. Thus, our NiO_x_-based sample device exhibits a smaller slope ${\text{of }}1.2 K_{B} T/q$ than that ($1.4 K_{B} T/q$) of the reference device, verifying that the NiO_x_-based device can efficiently reduce monomolecular recombinations with less charge carrier trapping, contributing to the improvement of the device performance^59^.

Further, we estimated *V*_bi_ of the perovskite photodiode using the relationship ($$V_{bi} = {\raise0.7ex\hbox{${ - nK_{B} T}$} \!\mathord{\left/ {\vphantom {{ - nK_{B} T} q}}\right.\kern-\nulldelimiterspace} \!\lower0.7ex\hbox{$q$}}\left( {\ln J_{0} } \right))$$ of the diode model^60^. The estimated value of $$V_{bi} {\text{is}} \approx 0.52 {\text{V}}$$ for the sample device with NiO_x_, while the value of $$V_{bi}$$ is ≈ 0.36 V for the reference device with PEDOT:PSS. Such a high $$V_{bi}$$ value of the sample device is a direct signature of the improved charge-selecting properties of the NiO_x_ HTL. Thus, it is clear that the hole-selecting capability of NiO_x_ is much greater than that of PEDOT:PSS, causing the high responsivity of the self-powered perovskite photodiode to be greater with NiO_x_^29^.

Next, to gain a deeper understanding of the charge transfer in the perovskite photodiodes based on distinct HTLs, impedance spectroscopy (IS) measurements were taken for the photodiodes in the dark. Figure 6c shows Nyquist plots from IS data for the photodiodes with NiO_x_ and PEDOT:PSS at zero bias voltage. We observed clear semicircles that distinguish the intermediate-frequency (*f*) regions for both photodiodes. These are linked to the charge transfer at the HTL/perovskite/ETL interfaces, primarily owing to recombinations. From the fitting of the IS data, the capacitance (*C*_1_), parallel resistance (*R*_1_), and series resistance (*R*_2_) values of the photodiodes with NiO_x_ and PEDOT:PSS were deduced. The equivalent circuit diagram is shown in the inset figure. The obtained values of *C*_1_, *R*_1,_ and *R*_2_ are correspondingly 3.87 nF, 87.04 Ω and 34.94 Ω for the sample device with NiO_x_ and are 3.90 nF, 95.48 Ω and 35.14 Ω for the reference device with PEDOT:PSS. The *C*_1_ and *R*_2_ values of the sample device are slightly lower than those of the reference device, indicating fewer surface trap state charges with less charge accumulation at the interface between the perovskite layer and the NiO_x_ HTL compared to the PEDOT:PSS HTL. Moreover, the *R*_1_ value of the sample device is clearly lower than that of the reference device. This low *R*_1_ of the NiO_x_-based perovskite photodiode can be attributed to the low interfacial charge transfer resistance at the perovskite/NiO_x_ interface. The smaller value of *R*_1_ also implies faster hole transport at the NiO_x_/perovskite interface compared to that at the PEDOT:PSS/perovskite interface.

Next, we also measured the temporal responses of the sample device with NiO_x_ as the HTL at various input power levels (*P* = 3, 69, 139, 209, 279, and 349 mW cm^−2^) while turning the incident laser light (532 nm) on and off (Fig. 6d). The rapid rise ($\tau_{r } )$ and decay ($\tau_{d }$) response times of the sample device were found to be $\tau_{r } \approx 0.9$ and $\tau_{d } \approx 1.8\,{\text{ms}},$ respectively, at zero bias voltage. For comparison, we also measured the photoresponse of the reference device (Fig. S8). From the comparison, it was found that the photoresponse of the sample device is much faster than that $\left( {\tau_{r} \approx 3, \tau_{d} \approx 10 \,{\text{ms}}} \right)$ of the reference device. Thus, it is clearly shown that the NiO_x_ HTL is a key component required to realise a fast self-powered perovskite-based photodiode.

**Figure 7:**
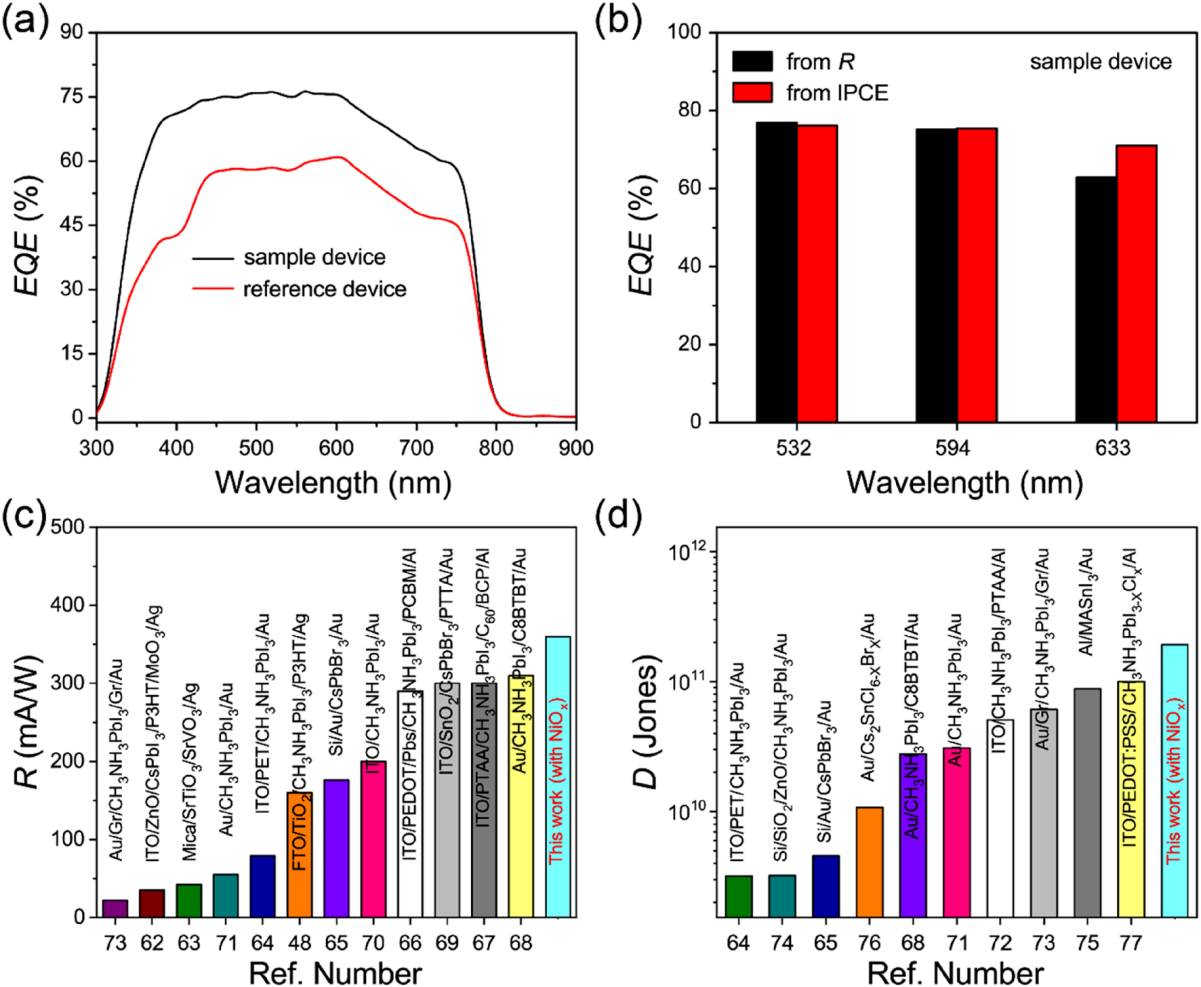
(**a**) *EQE* spectra as a function of the wavelength for the perovskite photodiodes with NiO_x_ or PEDOT:PSS as HTLs, (**b**) comparison of measured (from IPCE) and estimated (from *R*) values of the *EQE*s at zero bias voltage for three different wavelengths of incident light, and comparisons of the *R* (**c**) and *D* (**d**) values of several previously reported perovskite photodiodes.

Next, the *EQE*s of the perovskite photodiodes were also estimated from their *R* values using the following relationship^61^,

7 $$EQE = R\frac{hc}{{e\lambda }},$$

where *h* and *c* correspondingly denote the Planck constant $\left( {6.62 \times 10^{ - 34} \,{\text{J}}\,{\text{s}}} \right)$ and the speed of light $\left( {3.8 \times 10^{8} \,{\text{ms}}^{ - 1} } \right)$. The estimated *EQE* values are 76.5% and 61% for the sample device with NiO_x_ and the reference device with PEDOT:PSS, respectively, at a wavelength of 532 nm. As expected, the *EQE* value of the sample device with NiO_x_ is much higher than that of the reference device with PEDOT:PSS. This high *EQE* value for the sample device is the result of the improved charge-selecting properties as well as the improved exciton dissociation at the perovskite/HTL interface with the help of the high built-in electric field in the heterostructure. In order to confirm the estimated *EQE* values, we also measured the *EQE* spectra using an incident photon-to-current collection efficiency (IPCE) system, as shown in Fig. 7a. The measured *EQE* values from IPCE measurements were 76% and 60% for the sample and reference devices, respectively, at the wavelength of 532 nm, which are nearly identical to those estimated from the *R* values. For further comparison with other wavelengths, Fig. 7b shows the high *EQE* values estimated from both *R* and IPCE measurements of the sample device with NiO_x_, verifying the close correspondence of these values and supporting the high device performance of the perovskite photodiode with the NiO_x_ HTL.

Finally, the improved responsivity *R* value of our sample device is compared with those in previous reports to emphasise the novelty of the results with the electron-blocking NiO_x_ HTL (see Fig. 7c and Table S1)^12,48,62–71^. We also compared *D* with other reported values in Table S1, as shown in Fig. 7d^50,65,68,71–77^. It is clear from these figures that *R* and *D* of our photodiode with NiO_x_ in this study are much higher compared to those reported previously. These findings overall show that the interface quality and recombination activities in perovskite photodiodes are mainly influenced by the electron-blocking NiO_x_ HTL, which will surely provide a platform for further improvements in the performance of photodiodes coupled with excellent charge-selecting layers.

## Conclusions

In summary, we designed a self-powered hybrid organic–inorganic perovskite photodiode with an effective and capable electron-blocking hole-transport NiO_x_ layer. We used NiO_x_ as the HTL in the fabrication of the perovskite photodiode to improve the interface quality by suppressing the dark leakage current reaching to $7.84 \times 10^{ - 6} \,{\text{mA}}\,{\text{cm}}^{ - 2}$. The perovskite photodiode fabricated with NiO_x_ exhibited better PV performance with a PCE of 13% as compared to the photodiode with conventional PEDOT:PSS. A remarkably high responsivity *R* value of $360\,{\text{mA}}\,{\text{W}}^{ - 1}$ with detectivity $D = 2.08 \times 10^{11} {\text{Jones}}$ and $EQE = 76.5\%$ for the self-powered perovskite photodiode with the NiO_x_ HTL was noted under incident laser light with a wavelength of 594 nm at zero bias voltage. Further, the performance capabilities of the perovskite photodiode were estimated at different bias voltages; the value of *R* increased gradually to $428\,{\text{mA}}\,{\text{W}}^{ - 1}$ with $D = 3.6 \times 10^{11} {\text{Jones}}$ and $EQE = 77\%$ at a bias voltage of *V*_bias_ = − 1 V. Based on the diode model, we deduced an ideality factor of 1.1 and a high built-in potential value of $V_{bi} \approx 0.52 {\text{V}}$ for the photodiode with the NiO_x_ HTL, thus providing direct evidence of the improvement of the charge-selecting characteristics of the NiO_x_ layer. Furthermore, we observed fast rise and decay times of approximately 0.9 to 1.8 ms, respectively, for the perovskite photodiode with the NiO_x_ HTL at zero bias voltage, values which are much faster than those of the reference photodiode with the conventional PEDOT:PSS HTL. Therefore, the self-powered perovskite photodiode studied here opens up an opportunity for applications of hybrid perovskite heterostructures with the electron-blocking NiO_x_ HTL in highly sensitive light-detecting optoelectronic devices that consume low amounts of energy, such as optical sensors, waveguide-integrated photodiodes, and/or nano-photodetectors.

## Experimental section

### Materials

All materials (organic and inorganic) used here were obtained from commercial sources and were used without any extra purification steps. The materials of anhydrous gamma-butyrolactone (GBL, 99%), anhydrous dimethyl sulfoxide (DMSO, 99%), anhydrous chlorobenzene (CB, 99.9%), and nickel (II) nitrate hexahydrate (Ni(NO_3_)_2_·6H_2_O) (99.998%) were purchased from Sigma-Aldrich. Isopropyl alcohol (IPA, 99.7%) and ethylene glycol (C_2_H_6_O_2_, 99%) were purchased from Daejung. Lead (II) iodide (PbI_2_, 99%) was purchased from Alfa Aesar and the methyl-ammonium iodide (MAI, > 99%) used here was from Greatcell Solar. The suspension of ZnO NPs was purchased from Avantama (N-10). Other chemicals, specifically poly (3, 4-ethylene dioxythiophene) polystyrene sulfonate (PEDOT:PSS), were from H. C. Starck (Clevios PVP AI 4083). Phenyl-C61-butyric acid methyl ester (PCBM_60_) and bathocuproine (BCP, 98%) were purchased correspondingly from Nano-C and TCI.

### Device fabrication and characterisation

Glass substrates coated with an indium tin oxide (ITO) layer (80 nm, 30 Ω/square) were sequentially put into ethanol, a detergent, and deionised water (DI) in an ultrasonic bath (20 min for each step) to clean them. Subsequently, the ITO substrates were dried with N_2_ and treated with atmospheric O_2_ plasma for 2 min. A NiO_x_ precursor solution was prepared by dissolving Ni(NO_3_)_2_ 6H_2_O (≈ 0.029 g) in a mixed solvent of ethylene glycol and IPA at a 3:2 volume ratio.

To form the HTLs of NiO_x_ (40 nm) and PEDOT:PSS (40 nm), the prepared solutions were spin-coated onto ITO substrates at 3000 rpm for 40 s and at 4000 rpm for 35 s, respectively. For NiO_x_, the coated precursor layer was annealed at 70 °C for 1 min and then at 300 °C for 1 h, while for PEDOT:PSS, the coated layer was annealed at 120 °C for 30 min. To make the perovskite precursor solution, a mixture of MAI and PbI_2_ at a 1:1 molar ratio was dissolved in a mixed solvent of GBL and DMSO at a 7:3 volume ratio. The precursor solution was stirred overnight at 60 °C. The perovskite precursor solution (50 µl) was spin-coated at 1000 rpm for 10 s and then at 3000 rpm for 25 s to obtain the desired thickness of 250 nm onto the NiO_x_ and PEDOT:PSS layers. During the spinning process, an anti-solvent (toluene ~ 150 µl) was poured onto the precursor layer at 17 s. The perovskite precursor layer was then annealed at 100 °C for 20 min. For the ETL, a 20 mg/ml of PCBM_60_ solution, dissolved in CB, was spin-coated at 1500 rpm for 60 s onto the perovskite layer. A dispersion of ZnO NPs mixed with IPA (7:3) was then spin-coated at 1000 rpm for 10 s and at 4000 rpm for 40 s onto the PCBM_60_ layer. Finally, BCP (12 nm) and Al (70 nm) were deposited onto the ZnO NP layer using a thermal evaporation system. Thus, the device structure of the sample photodiode was [ITO/NiO_x_/CH_3_NH_3_PbI_3_/PCBM_60_/ZnO NPs/BCP/Al]. The active area of the fabricated devices was 6 mm^2^ in size.

### Film and device characterisation

The water contact angles of the fabricated NiO_x_ and PEDOT:PSS layers were measured using a contact angle meter (Phoenix 300 Touch, Surface Electro Optics). The fabricated perovskite film was characterised using a field emission scanning electron microscope (SEM, Model JSM-6700F, JEOL Co.) to analyse the surface morphology. To investigate the surface roughness and surface potential of the organic and inorganic functional layers, atomic force microscopy (AFM) and Kelvin probe force microscopy (KPFM, FlexAFM, Nanosurf AG) were used. To measure the surface potential, a Pt/Ir-coated silicon tip (resonance frequency = 87 kHz and a force constant = 3.9 Nm^−1^, NanoWorld, Inc.) was used while applying AC voltage of 1 V at a frequency of 18 kHz.

An X-ray diffractometer (XRD-Rigaku D/Max 2200, *λ* = 0.154 nm) was used to check the crystallinity of the perovskite layers on the NiO_x_ and PEDOT:PSS layers. To estimate the optical absorption spectra of the perovskite layers, a UV–visible spectroscopy system (8453, Agilent) was employed. The photocurrent versus bias voltage (*J*–*V*) characteristics were measured using a source meter (2400, Keithley) under illumination of incident laser light with several different wavelengths and were calibrated using a reference commercial silicon photodiode (THORLABS-PDA10A2) (see Fig. S9). The PV performance of the fabricated photodiode was measured under an illumination intensity of 100 mW cm^−2^ generated by an AM1.5 light source (Newport, 96,000 Solar Simulator) and calibrated using a reference cell (Bunkoh-keiki, BS-520). Impedance spectroscopy (IS) measurement of the photodiode was performed in the dark using an impedance analyser (HP 4192A) with an AC oscillating amplitude of 100 mV (RMS) to maintain the linearity of the response. The *EQE* spectra were also measured using an incident photon-to-current collection efficiency (IPCE) measurement system (IQE-200 EQE/IQE, Newport).

## Supplementary Information

Supplementary Information.
