# Supplementary material for: Highly efficient self-powered perovskite photodiode with an electron-blocking hole-transport NiOx layer
Source: Sci Rep. 2021 Jan 8;11:169. doi: 10.1038/s41598-020-80640-3 (PMC7794468; doi:10.1038/s41598-020-80640-3)
Supplement: Supplementary file 1 — Supplementary Information. [file 41598_2020_80640_MOESM1_ESM.docx]

Supporting Information

Highly Efficient Self-powered Perovskite Photodiode with an Electron-blocking Hole-transport NiO_x_ Layer

Amir Muhammad Afzal^a^, In-Gon Bae ^a^, Yushika Aggarwal^a^, Jaewoo Park^a^, Hye-Ryeon Jeong^a^, Eun Ha Choi^a^, and Byoungchoo Park*^a^

^a^Department of Electrical and Biological Physics, Kwangwoon University, Wolgye-Dong, Seoul 01897, South Korea

E-mail: bcpark@kw.ac.kr


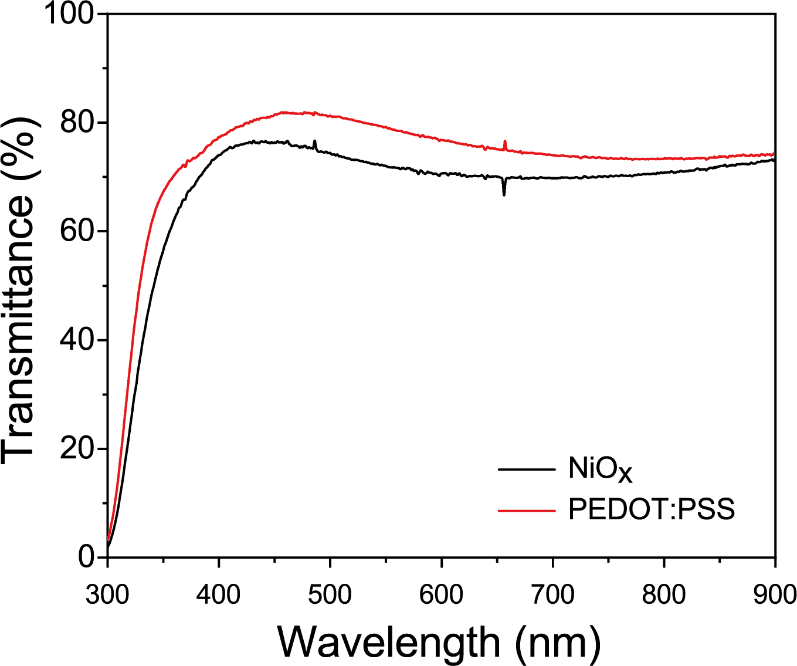


**Figure S1**. Optical transmittance outcomes of NiO_x_ and PEDOT:PSS layers

**
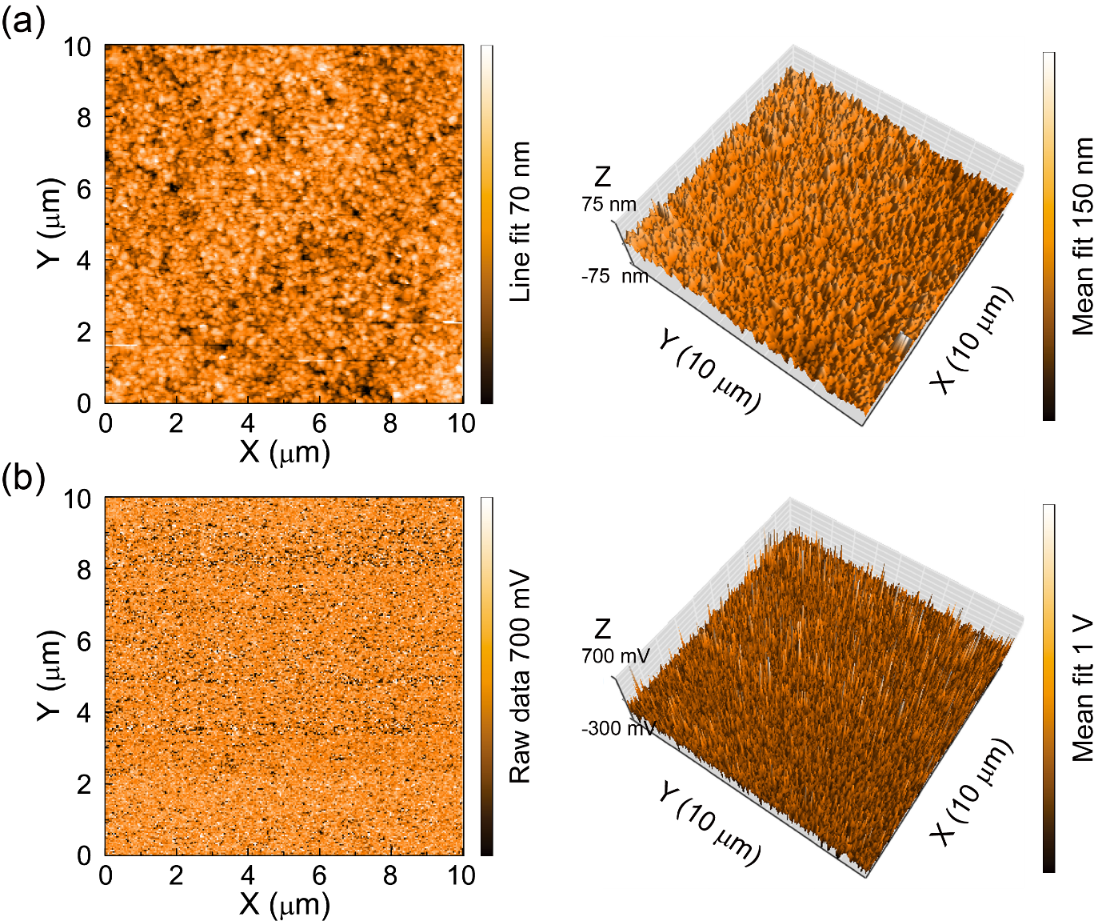
**

**Figure S2**. (**a**) AFM image of a perovskite film on a PEDOT:PSS layer (left) with a three-dimensional plot (right) to measure the roughness of the perovskite film on the PEDOT:PSS layer, and (**b**) KPFM image of the perovskite film on the PEDOT:PSS layer as a HTL (left) to measure the work function with a three-dimensional plot (right) to assess the potential difference of the perovskite film on the PEDOT:PSS layer


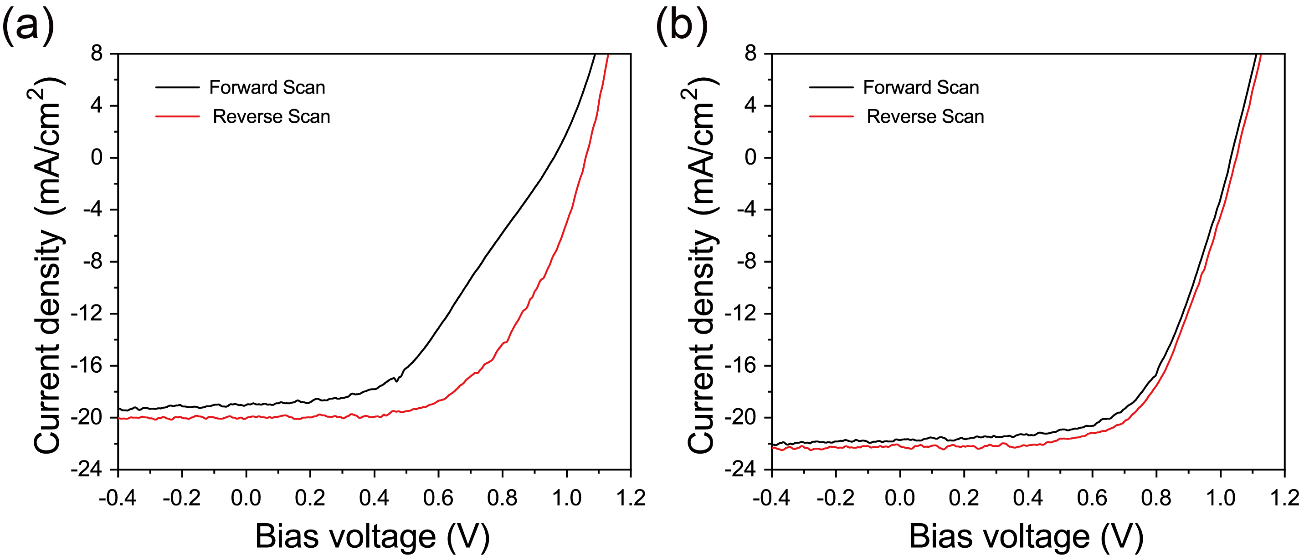


**Figure S3.** Forward and backward *J*-*V* scans of perovskite photodiodes with PEDOT:PSS (a) and NiO_x_ (b) layers as HTLs


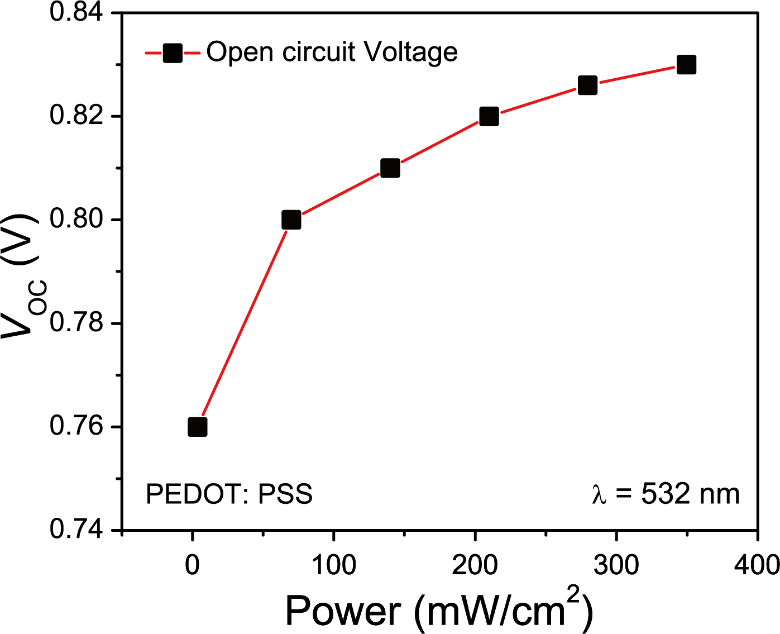


**Figure S4**. Open-circuit voltage of a perovskite photodiode with a PEDOT:PSS layer as a HTL

**
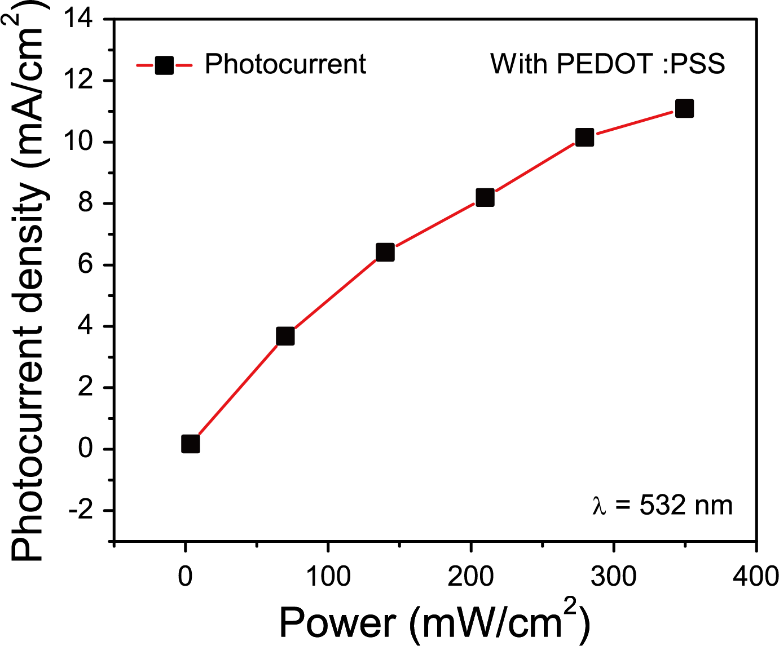
**

**Figure S5**. Photocurrent density of the perovskite photodiode with the PEDOT:PSS layer as a HTL at zero bias voltage


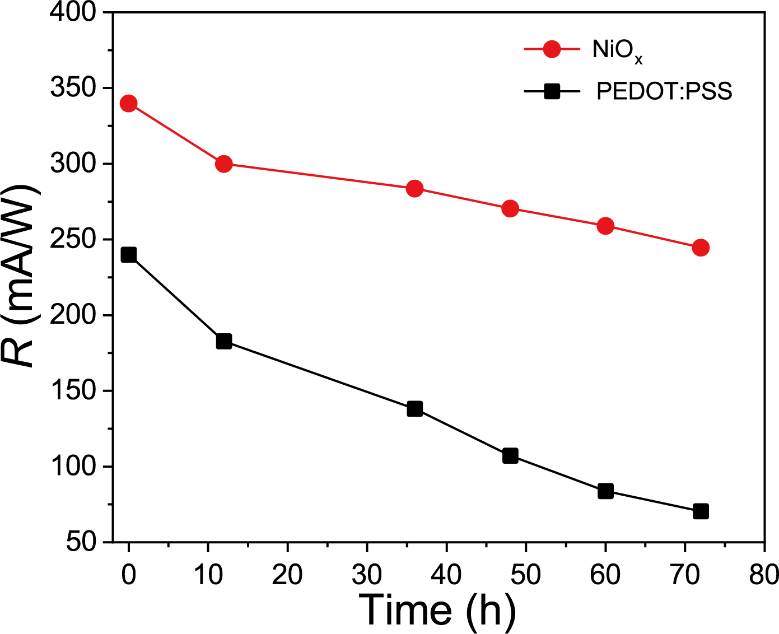


**Figure S6.** Stability characteristics of perovskite photodiodes with PEDOT:PSS and NiO_x_ layers as HTLs


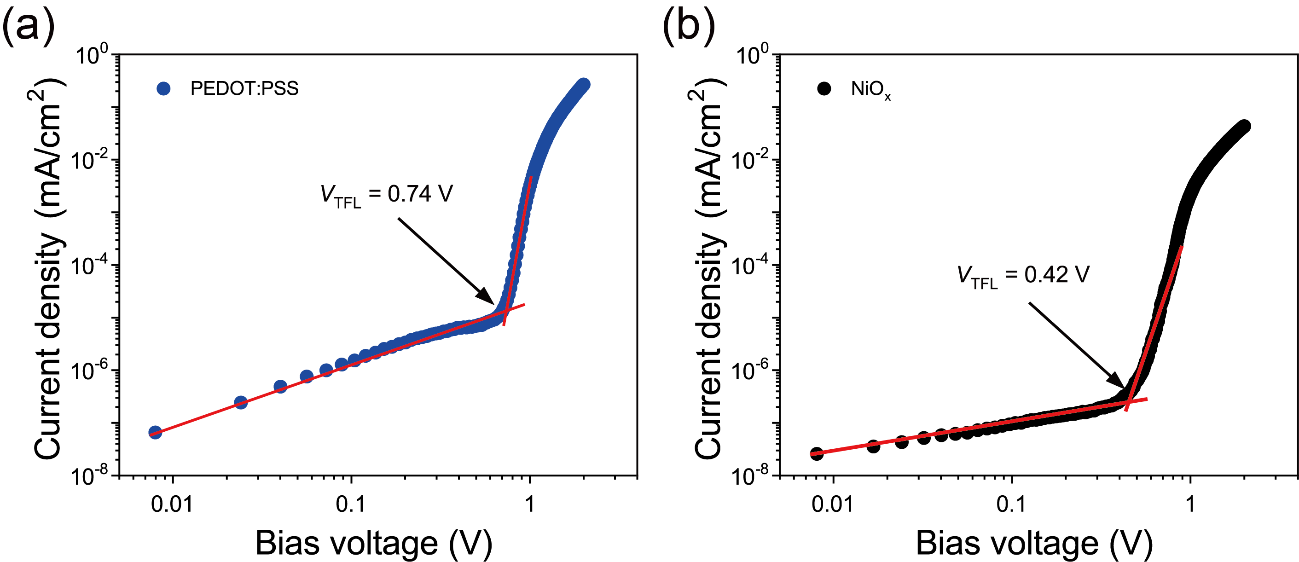


**Figure S7.** Dark *J–V* characteristics of devices with PEDOT:PSS (a) and NiO_x_ (b) layers as HTLs


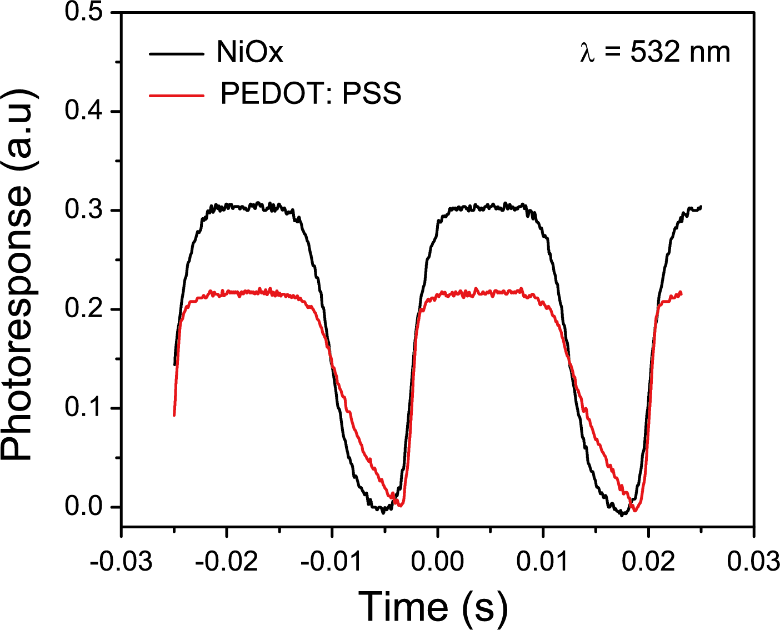


**Figure S8**. Comparison of the temporal responses of perovskite photodiodes with NiO_x_ and PEDOT:PSS layers as HTLs


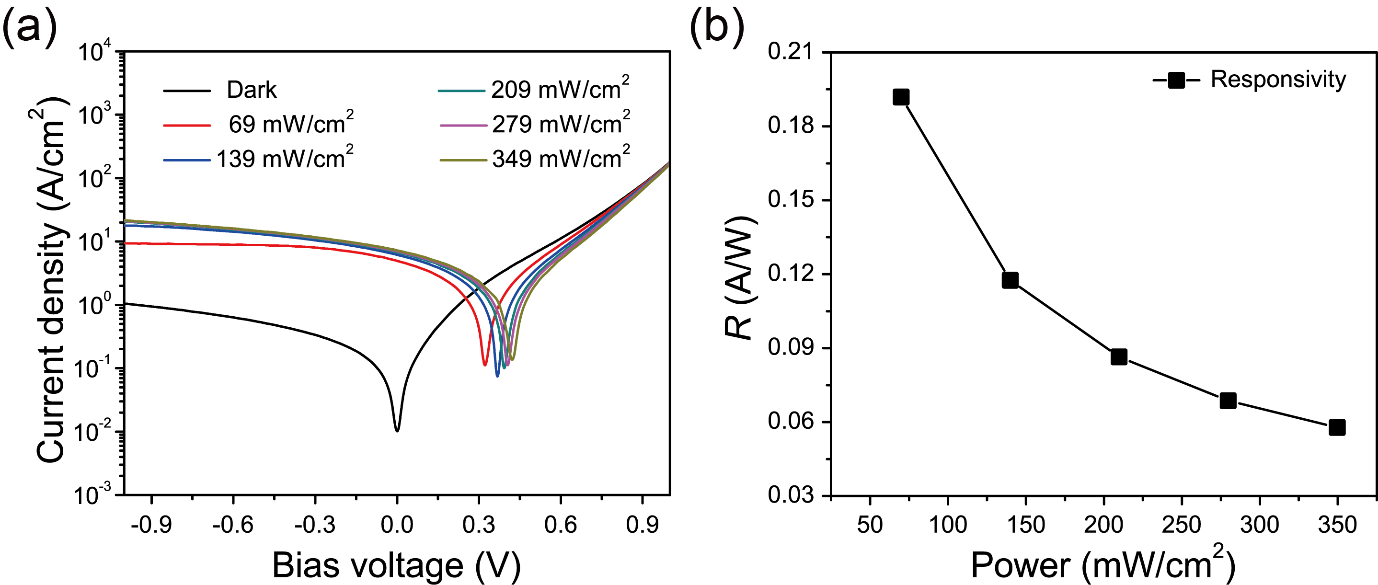


**Figure S9**. (**a**) Current density vs. applied bias voltage (*J-V*) characteristics for a commercial silicon photodiode as a reference to confirm our photodiode measurement system under incident laser light with a wavelength of 532 nm and with different power intensity levels, and (**b**) estimated responsivity *R* of a silicon photodiode as a function of the input power intensity

In order to confirm our photodiode measurement system, a commercial silicon photodiode (THORLABS-PDA10A2) was used as a reference. Before the measurement of the perovskite photodiodes, the reference silicon photodiode was used to measure the dark/photo *J-V* characteristics via the measurement system. The observed *J*-*V* characteristics of the silicon photodiode as a function of the input power of incident laser light are shown in Fig. S9a. Further, the *R* values (0.19 $AW^{-1}$) of the silicon photodiode were calculated, indicating self-consistency with the reported value (*R* $\approx$ 0.19 $AW^{-1}$) for the silicon photodiode (Fig. S9b).

Table S1. Comparison of perovskite photodiodes with NiO_x_ with previously reported outcomes.

| Type | Structure | $\boldsymbol{\lambda}$  (nm) | Bias  voltage  (V) | *R*  (mAW^-1^) | *D*  (Jones) | EQE  (%) | $\boldsymbol{\tau}_{\boldsymbol{R}}$/$\boldsymbol{\tau}_{\boldsymbol{D}}$  (ms) |
| --- | --- | --- | --- | --- | --- | --- | --- |
| Lateral | Au/CH_3_NH_3_PbI_3_/RhB/Au[^1^](#_ENREF_1) | 550 | 5 | 40 | - | 10 | 4 x 10^4^  /6 x 10^4^ |
|  | Si/CsPbBr_3_/Au[^2^](#_ENREF_2) | 532 | 2 | 180 | 6.1 x 10^10^ | - | 2.1 x 10^-1^  /1.3 x 10^0^ |
|  | Si/SiO_2_/Gr/ CH_3_NH_3_PbI_3_/Gr[^3^](#_ENREF_3) | 452 | 2 | 20 | 3.5 x 10^9^ | - | 8.7 x 10^1^  /5.4 x 10^2^ |
|  | Si/SiO_2_/CH_3_NH_3_PbI_3_/Go/Au[^4^](#_ENREF_4) | 520 | 5 | 70 | - | - | 3.8 x 10^3^  /9.4 x 10^3^ |
|  | Ag/CsPbBr_3_/CuI^[5](#_ENREF_5)^ | 540 | 0  (self-powered) | 1.4 | 6.2 x 10^10^ | - | 9.0 x 10^-1^  /1.8 x 10^0^ |
|  | ITO/C_s_PbSn_(1-y)_(I_y_Br_1 y_)/ITO^[6](#_ENREF_6)^ | 473 | 0  (self-powered) | 4.5 | 2.0 x 10^10^ | - | 4.25 x 10^0^  /4.82 x 10^0^ |
| Vertical | FTO/ CH_3_NH_3_PbBr_3_/Au[^7^](#_ENREF_7) | 450 | 0  (self-powered) | 10 | - | 3.17 | 2.3 x 10^3^  /2.7 x 10^3^ |
|  | FTO/ZnO/CH_3_NH_3_PbI_3_/MoO_3_/Au[^8^](#_ENREF_8) | 500 | 0  (self-powered) | 150 | 6.0 x 10^11^ | - | 2 x 10^3^  /2 x 10^3^ |
|  | FTO/C_60_/CH_3_NH_3_PbI_3_/GaN/In[^9^](#_ENREF_9) | 500 | 0.2 | 190 | 4.8 x 10^11^ | - | 3.4 x 10^2^  /5.9 x 10^2^ |
|  | Mica/SrTiO_3_/SrVO_3_//Ag[^10^](#_ENREF_10) | 532 | 0  (self-powered) | 40 | - | - | 3.9 x 10^0^  /1.2 x 10^0^ |
|  | Si/Au/Ti/ CH_3_NH_3_PbCl_3_/Pt[^11^](#_ENREF_11) | 365 | 15 | 40 | 1.2 x 10^10^ | - | 2.4 x 10^1^  /6.2 X10^1^ |
|  | PEN/AuNW/PEDT:PSS/ CH_3_NH_3_PbI_3_/PCBM/Al[^12^](#_ENREF_12) | 600 | -1 | 300 | - | 60 | 4.0 x 10^-3^  / 5.2 x 10^-3^ |
|  | ITO/PET/CH_3_NH_3_PbI_3_/Au[^13^](#_ENREF_13) | - | 1 | 70 | 1.2 x 10^13^ | - | 8.0 x 10^1^  / 8.0 x 10^1^ |
|  | ITO/ZnO/CsPbI_3_/P3HT/MoO_3_/Ag[^14^](#_ENREF_14) | 532 | 0.5 | 30 | 1.8 x 10^12^ | 7 | - |
|  | ITO/ CH_3_NH_3_PbCl_3_/PTAA/Al[^15^](#_ENREF_15) | 398 | -1 | 70 | 6.8 x 10^10^ | 25 | - |
|  | ITO/PTAA/ CH_3_NH_3_PbI_3_/C_60_/BCP/Cu[^16^](#_ENREF_16) | 540 | -0.3 | 260 | 1.0 x 10^12^ | 62 | 1.0 x 10^-4^  /3.0 x 10^-4^ |
|  | ITO/PEDT:PSS/Pbs/ CH_3_NH_3_PbI_3_/PCBM/Al[^17^](#_ENREF_17) | 600 | -2 | 300 | 5.0 x 10^12^ | 70 | - |
|  | ITO/SnO_2_/CH_3_NH_3_PbI_3_/C[^18^](#_ENREF_18) | 473 | 0  (self-powered) | 260 | 7.0 x 10^11^ | - | 8.0 x 10^-4^  /5.8 x 10^-3^ |
|  | FTO/MgO/CH_3_NH_3_PbI_3_/C[^19^](#_ENREF_19) | 473 | 0  (self-powered) | 59 | 1.5 x 10^12^ | - | 6.3 x 10^-1^  /1.6 x 10^0^ |
|  | Si/CH_3_NH_3_PbI_3_/Au[^20^](#_ENREF_20) | 405 | -1 | 13.6 | 5.9 x 10^10^ | - | 5.2.0 x 10^-4^ /2.4 x 10^-3^ |
|  | Au/CsPbBr_3_/CsPbI_3_/Au[^21^](#_ENREF_21) | 650 | 0  (self-powered) | 125 |  | - | 4.0 x 10^-2^ /2.96 x 10^0^ |
|  | ITO/NiO/CH_3_NH_3_PbI_3_/PCBM_60_/ZnO NPs/ BCP/Al  (This work) | 532  594  633 | 0  (self-powered) | 340  360  330 | 1.9 x 10^11^  2.0 x 10^11^  1.8 x 10^11^ | 75  76  70 | 9.0 x 10^-1^  /1.8 x 10^0^ |
|  | ITO/NiO/CH_3_NH_3_PbI_3_/PCBM_60_/ZnO NPs/ BCP/Al  (This work) | 532  594  633 | -1 | 440  428  436 | 3.2 x 10^11^  3.6 x 10^11^  3.5 x 10^11^ | 78  77  76 |  |

References

1 Teng, C.-j. *et al.* Organic dye-sensitized CH_3_NH_3_PbI_3_ hybrid flexible photodetector with bulk heterojunction architectures. *ACS Appl. Mater. Interfaces* **8**, 31289-31294 (2016).

2 Dong, Y. *et al.* Improving all‐inorganic perovskite photodetectors by preferred orientation and plasmonic effect. *Small* **12**, 5622-5632 (2016).

3 Chen, Z. *et al.* Improving Performance of Hybrid Graphene–Perovskite Photodetector by a Scratch Channel. *Adv. Electron. Mater.* **5**, 1900168 (2019).

4 He, M. *et al.* Chemical decoration of CH_3_NH_3_PbI_3_ perovskites with graphene oxides for photodetector applications. *Chem. Commun.* **51**, 9659-9661 (2015).

5 Zhang, Y., Li, S., Yang, W., Joshi, M. K. & Fang, X. Millimeter-sized single-crystal CsPbrB_3_/CuI heterojunction for high-performance self-powered photodetector. *J. Phys. Chem. lett.* **10**, 2400-2407 (2019).

6 Tang, X. *et al.* All-Inorganic Halide Perovskite Alloy Nanowire Network Photodetectors with High Performance. *ACS Appl. Mater. Interfaces* **12**, 4843-4848 (2020).

7 Cao, M. *et al.* Perovskite heterojunction based on CH_3_NH_3_PbBr_3_ single crystal for high-sensitive self-powered photodetector. *Appl. Phys. Lett.* **109**, 233303 (2016).

8 Zhou, H. *et al.* Ga-doped ZnO nanorod scaffold for high-performance, hole-transport-layer-free, self-powered CH_3_NH_3_PbI_3_ perovskite photodetectors. *Sol. Energy Mater. Sol.* **193**, 246-252 (2019).

9 Zhou, H., Mei, J., Xue, M., Song, Z. & Wang, H. High-stability, self-powered perovskite photodetector based on a CH_3_NH_3_PbI_3_/GaN heterojunction with C_60_ as an electron transport layer. *J. Phys. Chem. C* **121**, 21541-21545 (2017).

10 Xu, R. *et al.* Perovskite Transparent Conducting Oxide for the Design of a Transparent, Flexible, and Self-Powered Perovskite Photodetector. *ACS Appl. Mater. Interfaces* **12**, 16462-16468 (2020).

11 Maculan, G. *et al.* CH_3_NH_3_PbCl_3_ single crystals: inverse temperature crystallization and visible-blind UV-photodetector. *J. Phys. Chem. lett.* **6**, 3781-3786 (2015).

12 Bao, C. *et al.* Highly flexible self-powered organolead trihalide perovskite photodetectors with gold nanowire networks as transparent electrodes. *ACS Appl. Mater. Interfaces* **8**, 23868-23875 (2016).

13 Leung, S. F. *et al.* A self‐powered and flexible organometallic halide perovskite photodetector with very high detectivity. *Adv. Mater.* **30**, 1704611 (2018).

14 Sim, K. M., Swarnkar, A., Nag, A. & Chung, D. S. Phase Stabilized α‐CsPbI_3_ Perovskite Nanocrystals for Photodiode Applications. *Laser Photonics Rev.* **12**, 1700209 (2018).

15 Zheng, E., Yuh, B., Tosado, G. A. & Yu, Q. Solution-processed visible-blind UV-A photodetectors based on CH_3_NH_3_PbCl_3_ perovskite thin films. *J. Mater. Chem. C* **5**, 3796-3806 (2017).

16 Bao, C. *et al.* Low‐noise and large‐linear‐dynamic‐range photodetectors based on hybrid‐perovskite thin‐single‐crystals. *Adv. Mater.* **29**, 1703209 (2017).

17 Liu, C. *et al.* Ultrasensitive solution-processed broad-band photodetectors using CH_3_NH_3_PbI_3_ perovskite hybrids and PbS quantum dots as light harvesters. *Nanoscale* **7**, 16460-16469 (2015).

18 Pan, X. *et al.* Achieving a high-performance, self-powered, broadband perovskite photodetector employing MAPbI_3_ microcrystal films. *J. Mater. Chem. C* **8**, 2028-2035 (2020).

19 Yang, X. *et al.* MgO/ZnO microsphere bilayer structure towards enhancing the stability of the self-powered MAPbI_3_ perovskite photodetectors with high detectivity. *Appl. Surf. Sci.* **504**, 144468 (2020).

20 Geng, X. *et al.* Ultrafast Photodetector by Integrating Perovskite Directly on Silicon Wafer. *ACS Nano* **14**, 2860-2868 (2020).

21 Wang, M., Tian, W., Cao, F., Wang, M. & Li, L. Flexible and Self‐Powered Lateral Photodetector Based on Inorganic Perovskite CsPbI_3_–CsPbBr_3_ Heterojunction Nanowire Array. *Adv. Funct. Mater.* **30**, 1909771 (2020).
